# Developments in euthanasia practice in the Netherlands: Balancing professional responsibility and the patient’s autonomy

**DOI:** 10.1080/13814788.2018.1517154

**Published:** 2018-11-01

**Authors:** Pauline S. C. Kouwenhoven, Ghislaine J. M. W. van Thiel, Agnes van der Heide, Judith A. C. Rietjens, Johannes J. M. van Delden

**Affiliations:** a Julius Centre for Health Sciences and Primary Care, University Medical Centre Utrecht, Utrecht, The Netherlands;; b Department of Public Health, Erasmus Medical Centre, Rotterdam, The Netherlands

**Keywords:** Euthanasia, palliative and terminal care, health ethics, general practice/family medicine

## Abstract

In 2015, euthanasia accounted for 4.5% of deaths in the Netherlands, of which 93% were performed by a GP. Historically, a conflict of physician’s duties—to alleviate unbearable suffering and at the same time preserve the patient’s life—is central to the justification of euthanasia practice in the Netherlands. However, there seems to be a shift towards a greater emphasis on the patient’s autonomous wish as the primary basis for euthanasia. This shift has consequences for the role and interpretation of the physician’s duties in end-of-life care. This paper aims to describe these developments in euthanasia practice and end-of-life decision-making. We describe important relevant developments and look into the role and the meaning of two dimensions of the concept of ‘patient autonomy’ regarding end-of-life decisions, in particular, the euthanasia request. We claim that the concept of autonomy ‘as a right,’ which can be distinguished from autonomy ‘as an ideal,’ narrows the physician’s window of opportunity to offer end-of-life care other than euthanasia.

KEY MESSAGES

- There seems to be an increased emphasis on the autonomy of the patient as the basis for euthanasia in the Netherlands.
- (Re)discovering the right balance between the physician's professional responsibility and the patient's autonomy is essential for providing good end-of-life care.

## Introduction

Legalizing physician assistance in dying is an issue of debate in countries worldwide [1]. In the Netherlands, euthanasia and assisted suicide are punishable under Dutch criminal law unless a physician meets the criteria of due care stipulated by the Dutch euthanasia law (Box 1) and reports the procedure. In the Netherlands, euthanasia is performed mostly by general practitioners (GPs) (in 93% of cases in 2015) [2,3]. A conflict of physician duties—to alleviate unbearable suffering and at the same time preserve the patient’s life—is central to the justification of euthanasia practice [4–6]. The Royal Dutch Medical Association (KNMG) considers euthanasia and assisted suicide as the *ultimum remedium*, the last resort option for patients with unbearable suffering [5].

We, as authors and experts in the field of research as well as in experience in general practice, have reasons to believe that the approach to euthanasia as a practice based on the physician’s conflict of duties is shifting towards euthanasia as a practice grounded in the patient’s autonomous choice. In the past ten years, the number of cases of euthanasia has increased from 1.7% of deaths in 2005 to 4.5% in 2015 [3]. Further study is needed to understand the underlying causes fully [3]. However, in 80% of cases, the main reason for euthanasia is the patient’s wish or no prospect of improvement. This percentage is higher than for pain or other symptoms [4]. This could point an increasing demand of patients to control their end-of-life.

Box 1 The criteria of due care requires that the physician

(1) is convinced that there is a voluntary and well-considered request from the patient, and
(2) is convinced that the patient is suffering unbearably without the prospect of improvement, and
(3) has informed the patient about his current situation and prospects, and
(4) has come to the conclusion—together with the patient—that no reasonable alternative solution to alleviate the patient’s suffering exists, and
(5) has consulted at least one independent physician, who visited the patient personally and has given a written assessment of the criteria of due care, and
(6) has performed euthanasia or PAS with due medical care and attention.
From: Termination of Life on Request and Assisted Suicide (Review Procedures) Act (2002)

If the physician’s conflict of duties is no longer the primary justification for granting a euthanasia request, the physician faces a significant dilemma. It may be that the patient strongly desires euthanasia, but the physician feels granting the request would not comply with proper care. In such a situation, the physician has a professional judgement that does not involve a conflict of duties (yet). To clarify this dilemma and possible ways forward, we will relate two dimensions of the concept of autonomy to the role of the physician in end-of-life decision-making.

## Developments emphasizing the role of patient autonomy

### Criteria of due care and the code of practice

An indication for a growing emphasis on the role of the patient’s autonomous wish can be found in the ‘code of practice’ of the Dutch Euthanasia Review Committees [7]. This document guides the interpretation of the statutory criteria of due care (Box 1). According to the code of practice, suffering may comprise ‘anxiety about future deterioration’ and/or ‘be the result of a summation of mental and physical aspects’. This is a broad interpretation, which challenges the objective assessment of suffering, yielding a major role for subjective elements, mainly the patient’s experience. Furthermore, regarding the requirement of ‘no reasonable alternative solution,’ the code states, ‘The patient may refuse (palliative) care or treatments, which does not always have to stand in the way of granting the (euthanasia) request.’ An alternative solution may not be considered reasonable if the patient rejects it, even though in medical terms it may well have provided benefit. This way, the code of practice gives room for a shift towards a practice of euthanasia that is predominantly based on the patient’s autonomous choice.

### The role of the End-of-Life Clinic

In 2012, the ‘Dutch Right to Die Society’ (NVVE) founded the ‘End-of-Life Clinic.’ The Society’s key objective is to enhance freedom of choice at the end of life. It believes that the room the law offers for people who are suffering and want to end their life is not utilized sufficiently by physicians [8]. The aim of the End-of-Life Clinic, therefore, is to offer euthanasia—within the limits of the law—to people whose treating physician rejects their request for euthanasia or assisted suicide [9]. It has ambulant teams of physicians and nurses for this purpose. Their physicians are known to be less reticent regarding euthanasia in case of mental suffering than other Dutch physicians [10]. The End-of-Life Clinic seems to support and enhance the emphasis on the autonomous wish of the patient in euthanasia practice, because its essential aim is to grant a euthanasia request in case of unbearable suffering without the prospect of improvement, just as the euthanasia law permits [2]. However, if care is considered in a broader context than just fulfilling the patient’s autonomous wish to die, what fits into the law may not necessarily be the best possible care.

### Discussions regarding the euthanasia law and completed life

In 2010, a public initiative arose with the aim to legalize assistance in dying for elderly people who consider their life completed. It is explicitly directed at self-empowerment of the elderly [11]. Euthanasia or assisted suicide on the grounds of a completed life only, i.e. without serious illness, extends beyond the scope of the euthanasia law. In February 2016, an Advisory Committee on a Completed Life commissioned by the Ministry of Public Health published its report. The committee concluded that the current euthanasia law offers sufficient room to address most problems of older people who feel that their life is completed [12]. In its response, the government declared it wanted to create a separate legal framework for the (few) people whose life is completed and who have a wish to die without unbearable suffering due to any medical condition, and therefore, to enable the elderly to exercise autonomy for their death [13]. These developments show a change in society in the direction of more self-empowerment and even towards a right to die. This change was confirmed in the latest Dutch evaluation of the euthanasia law [14].

### Developments in public and professional opinions between 2010 and 2017

In 2010, the Dutch KOPPEL-study showed that most Dutch physicians and the public support the current legislation on euthanasia [15]. Both groups, however, also seemed reluctant to euthanasia when the patient’s suffering is of a psychological origin or nature [16]. Six years later, the third evaluation of the euthanasia law (2016) showed a similar picture as far as the opinions of physicians are concerned. However, there appears to be less reluctance among members of the public against euthanasia for non-somatic suffering (Table 1) [4].

**Table 1.** Opinions of physicians and the public about euthanasia and physician-assisted suicide.^a^

	KOPPEL (2010) Agree (%)		Third evaluation of the euthanasia law (2016) Agree (%)	
Vignettes	Physicians	Public	Physicians	Public
Do you personally agree with euthanasia or physician-assisted suicide in a case of a patient with…				
Cancer and loss of control, with severe pain	77	65	–	–
Cancer and loss of control, without physical symptoms	37	39	–	–
Old age and tired of living (completed life)	18	26	–	38
Severe depression	35	28	–	40
Early dementia and anxiety about the future	28	24	–	–
Advanced dementia, based on an advance euthanasia directive	33	85	–	83
In my opinion…				
… euthanasia should be allowed for people who are tired of living, without having a severe disease.	–	21	–	–
… the oldest old should be able to get medications that enable them, if they want so, to end their lives.	–	36	–	51
… everybody should have a right to euthanasia.	28	57	–	67
… every human being has the right to determine his or her own life and death.	58	53	59	49

a In the 2010 (KOPPEL) and 2016 (third evaluation of the euthanasia law) studies, questions were not always comparable; hence the table shows missing values.

## Autonomy as a right and as an ideal

Since the 1960s, ‘autonomy’ has become increasingly important in health law. This has resulted in an emphasis on one of the multiple dimensions of autonomy, i.e. autonomy understood as a right to freedom and self-determination. This is also referred to as ‘negative’ autonomy: freedom from external interference in one's own life [17,18]. The right of self-determination has since been increasingly emphasized as a fundamental right of a patient. This has led, for example, to the rule of 'informed consent’ in which the right to be informed about one’s diagnosis, prognosis and treatment options and the right to consent to medical treatment are combined as an expression of self-determination. Thus defined, autonomy forms a result of the right to privacy and the right to bodily integrity, as laid down in the European Convention for the Protection of Human Rights and in the Constitution.

If the emphasis is put on negative autonomy, input from other people about making (medical) decisions may be considered a threat rather than being helpful. Translated to the practice of euthanasia, the physician’s role could be viewed as entirely instrumental: the patient’s right to self-determination should be the primary consideration. That would imply that a doctor should grant a euthanasia request whenever this is possible within the law.

A second dimension of autonomy—complementary to autonomy understood as a right to freedom—is autonomy that functions as an ideal, which is sometimes referred to as ‘positive’ autonomy. The autonomous self is dynamic and constantly evolving. Achieving autonomy is a process in itself. Humans are social creatures who live life in interaction with others. Healthcare professionals should help patients to clarify what they want and to take control. The ideal of positive autonomy also resonates in the new definition of health: health as the ability to adapt and to self-manage, in the face of social, physical and emotional challenges [19]. The duties of a good healthcare professional are a supplement to the requirement of informed consent, to address the ideal of positive autonomy adequately. This presupposes a good relationship between healthcare professionals and patients and requires commitment, trust and good communication. Furthermore, complexities most often occur in the process towards granting a euthanasia request, and not during the actual performance of it. The course of that process is not only influenced by the patient, but also by the involved relatives. Excellent communication about expectations is of great importance in decision-making in end-of-life care [20].

The vulnerability and dependence of patients with a euthanasia request seem to call for care, support and a positive and relational concept of autonomy, rather than a negative concept of autonomy [18,21].

## The role of the physician

If the emphasis on patient autonomy ‘as a right’ is increasing, what might be the consequences for the role of the physician, most often the GP? Because of the described shift towards more autonomy-based euthanasia, treating physicians could get confused about the right approach to a patient requesting euthanasia. Should they, if possible, accept and follow the patient's wishes, or is it their professional responsibility to investigate, clarify and guide the euthanasia request and the suffering in a broader medical context? Moreover, is there enough room really to engage in a dialogue, now that the patient can turn to the End-of-Life Clinic? Patients may express that 'if you do not do it, I will find another physician who will' and this could undermine the relationship of trust between physician and patient. In such cases, where the request for euthanasia is not actually up for discussion, the physician is not given any real opportunity to use his or her knowledge and skills within an entrusted relationship and as a result, the patient may miss the opportunity of good palliative or psychosocial care. The real question or the actual problem that formed the basis for the euthanasia request may remain hidden in this way.

## A plea for a broader notion of autonomy

The previous analysis of the increased emphasis on patient autonomy ‘as a right’ and as the basis for euthanasia is relevant beyond Dutch borders. Physicians, especially GPs, in other countries should be aware of the importance of their professional role in end-of-life decision-making. Pressure on this role can create a void in which the doctor’s opportunity to offer and discuss alternatives to euthanasia is at risk of being lost. This may erode good end-of-life care. Even if autonomy is the predominant factor in an (incurably) ill patient's request for euthanasia, it still may be that there is an underlying question that requires a solution other than the ending of life [22,23]. That might be a need of care, attention, security, solace or some other way to decrease suffering.

A physician's refusal to grant a request for euthanasia, or reluctance to immediately agree with the proposal, seems to be regarded more and more as something related to the person of the physician—e.g. the physician’s outlook on life, or lack of experience, inclination or time—and less as something that has to do with a professional assessment of the patient's condition. Such a perspective on the physician’s role and responsibilities, based on a concept of autonomy ‘as a right’, differs from the perspective on the physician’s role in ordinary medical situations. Then shared decision-making—corresponding with autonomy ‘as an ideal’—is the preferred model; the patient generally trusts the physician's judgement, and patient and physician generally succeed in jointly deciding on an adequate approach. In end-of-life care it is central to the justification of euthanasia, that the physician experiences a conflict of duties between the duty to preserve the patient’s life and the duty to alleviate unbearable suffering. Physicians have the ethical responsibility to do no harm.

Physicians are faced with the task of finding the right balance between their professional responsibility and the patient's autonomy. If that balance is taken away, the physician risks to harm, and the patient risks missing the care that is needed [24]. A person requesting assistance in dying is much more than just his or her death wish. Even if a physician could meet all criteria of due care, granting a request for euthanasia is not by definition the best option for the patient.

## Conclusion

In the Netherlands, we observe a shift from ‘a conflict of duties’ of the physician to the autonomy of the patient as the basis for the practice of euthanasia. This societal development has consequences for the role and interpretation of the duties of the physician facing a euthanasia request, often a GP. The shift in emphasis towards the autonomy of the patient seems more than anything else to correspond to the approach of autonomy ‘as a right.’ However, patients who request euthanasia could benefit more from an approach of autonomy ‘as an ideal.’ The assessment and treatment of unbearable suffering by the physician are crucial in such an approach. Thus, the patient has the best chance of receiving the care he needs in a broader context. It is all about rediscovering the right balance between the physician's professional responsibility and the patient's autonomy.
